# Mycobacterium Growth Indicator Tube Time-To-Positivity Can Serve As an Early Biomarker of Treatment Response in *Mycobacterium avium* Complex Pulmonary Disease

**DOI:** 10.1016/j.chest.2021.08.046

**Published:** 2021-08-13

**Authors:** Rabi Danho, Jodie A. Schildkraut, Sanne M.H. Zweijpfenning, Elin M. Svensson, Lian J. Pennings, Saskia Kuipers, Heiman F.L. Wertheim, Martin J. Boeree, Wouter Hoefsloot, Jakko van Ingen

**Affiliations:** a Department of Medical Microbiology, Radboud University Medical Center for Infectious Diseases, Radboud University Medical Center, Nijmegen, The Netherlands; b Department of Pulmonary Diseases, Radboud University Medical Center for Infectious Diseases, Radboud University Medical Center, Nijmegen, The Netherlands; c Department of Pharmacy, Radboud University Medical Center for Infectious Diseases, Radboud University Medical Center, Nijmegen, The Netherlands; d Department of Pharmacy, Uppsala University, Uppsala, Sweden

To the Editor:

The recommended treatment regimen for *Mycobacterium avium* complex pulmonary disease (MAC-PD) consists of three drugs: rifampicin, ethambutol, and azithromycin. IV amikacin can be added in the initial 2 to 3 months for patients with severe or recurrent disease.^1^ With the recommended treatment regimens, 65% of treated patients attain culture conversion^2^; the median time-to-culture-conversion amounted to 4 (range, 1 to 12) months in a previous study.^3^ Because culture conversion requires multiple negative cultures that need to be incubated for 8 weeks, it takes up to 8 months to demonstrate culture conversion.^4^ Hence, there is a clear need for a biomarker to predict earlier on whether conversion will be reached or whether a change in treatment regimen is necessary to achieve it.

The use of (semi)quantitative culture analysis has been suggested as a relative rapid biomarker of treatment response.^3^^,^^5^ Yet the methods applied in the two existing studies, colony-forming unit counts and a semiquantitative scale, are labor-intensive and not available in routine laboratories. Clinical trials of TB treatment have shown clear correlations between *M TB* colony-forming unit counts and the time-to-positivity (TTP) of the widely used Mycobacterium Growth Indicator Tube (MGIT) automated broth culture system^6^; this correlation was also observed in a smaller study in MAC-PD.^7^

Here, we study the use of TTP in the MGIT automated broth culture system as a biomarker for treatment response in MAC-PD.

## Methods

Using current criteria, we performed a retrospective chart and laboratory database review of adult patients who received a diagnosis of macrolide-susceptible MAC-PD^1^ who received treatment for at least 6 months at Radboud University Medical Center Dekkerswald reference clinic from 2013 to 2019. Patients were excluded if no sputum culture result was available at baseline (treatment start date ±1 week), after 6 months (± 3 weeks) of antibiotic treatment, if less than three sputum cultures were performed or if no TTP data were available during the first 6 months of treatment. When multiple cultures with TTP data were available from the same timepoint during treatment, the average was used.

Demographic data, disease manifestation (fibrocavitary vs nodular-bronchiectatic), treatment regimen and culture status after 6 months of treatment and (if available) at the end of treatment were recorded from the electronic medical file and laboratory information system. We applied the NonTuberculous Mycobacteria - Network European Trials group (NTM-NET) outcome definitions for nontuberculous mycobacterial pulmonary disease (NTM-PD)^8^; for culture conversion, a more lenient definition was used: two consecutive negative cultures, collected at least 4 weeks apart. Conversion date was defined as the date of the first negative culture.^8^

Sputum samples were decontaminated with the N-acetyl-l-cysteine–sodium hydroxide method and concentrated by centrifugation before inoculation in MGIT automated liquid culture and on Lowenstein-Jensen solid medium. MGIT liquid cultures were incubated for 42 days.^4^ Isolated mycobacteria were identified as MAC with the use of the InnoLiPA Mycobacteria v2 (Innogenetics) line probe assay, which includes species-specific probes for *M avium*, *M intracellulare*, and *M chimaera.*^4^ Machine-generated TTP data of MGIT liquid culture were recovered from the laboratory information system and rounded to days to reflect differences in time between sputum expectoration and laboratory processing. For negative MGIT cultures, a TTP of 43 days was recorded. Statistical comparisons between groups were made using χ2 and *t*-tests, unless stated otherwise, with the use of SPSS software (version 25; IBM).

## Results

We included 49 patients; their baseline characteristics are presented in Table 1. After 6 months of therapy, 34 of 49 patients (69%) attained sputum culture conversion. Mean baseline TTP (with SD) was significantly different between converters and nonconverters (7.68 ± 4.64 vs 4.87 ± 2.20 days; *P* = .031) overall and in the 40 patients who were treated with three-drug regimens (ie, excluding those with most severe manifestations; 7.96 ± 5.07 vs 4.92 ± 2.36 days; *P* = .047). A baseline TTP of >7 days was associated with culture conversion (likelihood ratio, 6.947; *P* = .014) and a receiver operating characteristic curve identified a sensitivity of 41% and specificity of 93% to predict conversion with the use of the TTP >7 day cutoff.

**Table 1:** Characteristics and Results of the 49 Patients With *Mycobacterium avium* Complex Pulmonary Disease by Culture Conversion Status

Culture conversion	Yes (n = 34)	No (n = 15)	Total (N = 49)

Female patients, No. (%)	19 (56)	8 (53)	27 (55)
Age, mean ± SD, y	65.59 ± 9.32	60.53 ± 10.45	64.0 ± 9.9
Fibrocavitary disease, No. (%)	18 (53)	10 (67)	28
Nodular-bronchiectatic disease, No. (%)	16 (47)	5 (33)	21
*M avium*, No.	16	6	22
*M intracellulare*, No.	8	4	12
*M chimaera*, No.	10	5	15
Baseline time to positivity, mean ± SD, d	7.68 ± 4.64	4.87 ± 2.20	6.82 ± 4.23
Time to positivity after 3 mo, mean ± SD, d	36.38 ± 12.30	9.75 ± 5.19	32.57 ± 14.89
Samples per patient, mean ± SD, No.	5.32 ± 1.20	4.87 ± 1.46	5.18 ± 1.29
3-Drug regimen, No. (%)	27 (79)	13 (87)	40^a^
4/5-Drug regimen, No. (%)	7 (21)	2 (13)	9^b^
Cure at end of treatment, No. (%)	22 (65)	2 (13)	24 (49)

a Rifampicin-ethambutol-azithromycin (n = 25), clofazimine-ethambutol-azithromycin (n = 14), or rifampicin-ethambutol-clarithromycin (n = 1).

b Amikacin-clofazimine-rifampicin-ethambutol-azithromycin (n = 8), clofazimine-rifampicin-ethambutol-azithromycin (n = 1), or or amikacin-rifampicin-ethambutol-azithromycin (n = 1).

Mean baseline TTP was significantly different between patients with nodular-bronchiectatic disease and those with fibrocavitary disease (8.86 ± 5.62 vs 5.29 ± 1.65 days; *P* = .010) but did not differ between causative MAC species. Disease manifestation, patient age, and number of samples available for analysis were not associated significantly with culture conversion (*P* = .371; *P* = .095, and *P* = .256, respectively) (Table 1). Within the patients with fibrocavitary disease and nodular-bronchiectatic disease, mean baseline TTP differed between converters and nonconverters, but this difference was not statistically significant (5.72 ± 1.67 vs 4.50 ± 1.35 days [*P* = .059] in patients with fibrocavitary disease and 9.88 ± 5.86 vs 5.60 ± 3.44 days [*P* = .142] in patients with nodular-bronchiectatic disease).

**Figure 1:**
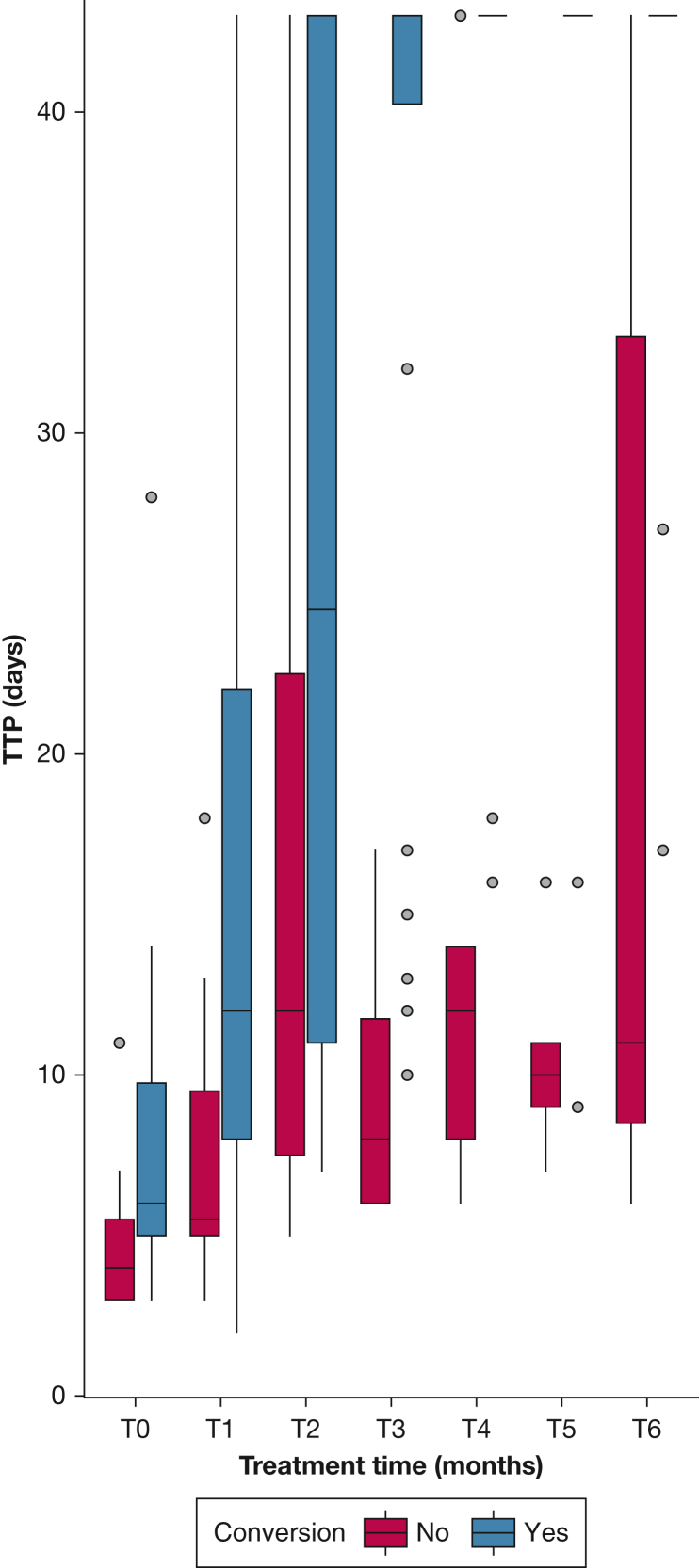
Liquid culture time-to-positivity over time in converters and nonconverters. Red indicates cohort of patients that did not attain culture conversion. Blue indicates cohort of patients that did attain culture conversion. TTP = time-to-positivity of liquid culture (in days).

Differences in TTP became larger after 3 months of treatment (36.38 ± 12.30 days in converters vs 9.75 ± 5.19 in nonconverters; *P* < .001) (Fig 1). A TTP >15 days after 3 months of treatment was associated with culture conversion (likelihood ratio, 5.365; *P* = .021) and the receiver operating characteristic curve identified a sensitivity of 83% and specificity of 75% to predict conversion with the use of a TTP cutoff of >15 days. The mean absolute difference (TTP at 3 months – TTP baseline) was 28.50 ± 11.96 days in converters and 4.50 ± 6.56 days in nonconverters (*P* = .001).

Baseline TTP was not significantly different among patients who experienced good outcome (cure; n = 24; 7.88 ± 5.29 days) vs those with bad outcome (failure, recurrence, halted, died; n = 25; 5.80 ± 2.61 days; *P* = .086) at the end of treatment; this correlation was also absent for TTP after 3 months of treatment (36.57 ± 12.85 days in those cured; 28.57 ± 16.16 days in those with bad outcomes; *P* = .160).

## Discussion

In our cohort, a baseline TTP of >7 days and TTP of >15 days after 3 months of treatment predicted that culture conversion would occur within the first 6 months of treatment (Fig 1). Our cohort characteristics and outcomes are in line with recent case series and reviews.^2^^,^^3^^,^^9^

Current guidelines recommend adding amikacin to treatment regimens in patients with severe disease or prior treatment^1^; clinical trials should establish whether the addition of amikacin should be based on bacterial load (as measured by TTP) rather than clinical and radiologic features. If our findings can be confirmed in prospective studies, TTP data after 3 months and the TTP changes from baseline until 3 months might allow shortening of treatment trials of new antibiotics for treatment of naïve patients to 3 months. This would substantially speed up the development of new, more effective treatment regimens in MAC-PD.

This study has important limitations, in line with the small-scale, retrospective, and uncontrolled nature; not all patients had cultures performed monthly, and those who did mostly submitted just one sputum sample. Patients also received different treatment regimens both in terms of choice and number of antibiotics. Furthermore, TTP data are censored if >42 days, which limits their analysis. Future studies ideally would derive their TTP data from controlled clinical trials.

MGIT TTP at baseline and in the first 3 months of MAC-PD treatment can be used to predict sputum culture conversion at 6 months. This early and easily available biomarker can be a useful tool in clinical practice and in trials that are evaluating new therapies. Its predictive value must be confirmed in larger scale controlled studies.
